# Exploring the role of flavin-dependent monooxygenases in the biosynthesis of aromatic compounds

**DOI:** 10.1186/s13068-024-02490-9

**Published:** 2024-03-22

**Authors:** Tong Shi, Xinxiao Sun, Qipeng Yuan, Jia Wang, Xiaolin Shen

**Affiliations:** State Key Laboratory of Chemical Resource Engineering, Beijing University of Chemical Technology, 15 Beisanhuan East Road, Chaoyang District, Beijing, 100029 China

**Keywords:** Hydroxylase, Protein engineering, Cofactors, Enzyme components

## Abstract

Hydroxylated aromatic compounds exhibit exceptional biological activities. In the biosynthesis of these compounds, three types of hydroxylases are commonly employed: cytochrome P450 (CYP450), pterin-dependent monooxygenase (PDM), and flavin-dependent monooxygenase (FDM). Among these, FDM is a preferred choice due to its small molecular weight, stable expression in both prokaryotic and eukaryotic fermentation systems, and a relatively high concentration of necessary cofactors. However, the catalytic efficiency of many FDMs falls short of meeting the demands of large-scale production. Additionally, challenges arise from the limited availability of cofactors and compatibility issues among enzyme components. Recently, significant progress has been achieved in improving its catalytic efficiency, but have not yet detailed and informative viewed so far. Therefore, this review emphasizes the advancements in FDMs for the biosynthesis of hydroxylated aromatic compounds and presents a summary of three strategies aimed at enhancing their catalytic efficiency: (a) Developing efficient enzyme mutants through protein engineering; (b) enhancing the supply and rapid circulation of critical cofactors; (c) facilitating cofactors delivery for enhancing FDMs catalytic efficiency. Furthermore, the current challenges and further perspectives on improving catalytic efficiency of FDMs are also discussed.

## Background

Aromatic compounds have found extensive use as active ingredients in nutraceuticals, cosmetics, and pharmaceuticals due to their valuable bioactivities, including antioxidant, anti-aging, antimicrobial, and antiviral activities [1–3]. These aromatic compounds exist in nature with various group modifications, such as hydroxylation, glycosylation, and methylation [4–8]. Among those modifications, the introduction of hydroxyl functional groups through hydroxylation serves as a prerequisite for glycosylation and methylation of certain aromatic compounds and plays a crucial role in their pharmacological activities [9, 10]. Numerous studies have highlighted the exceptional bioactivities of hydroxylated aromatic compounds. For instance, hydroxylation enhances the antimicrobial efficacy of aromatic compounds. Notably, just 30 μg/mL of 3,4,5-trihydroxybenzoic acid can inhibit the growth of certain gram-negative bacteria, whereas 3,4-dihydroxybenzoic acid and 4-hydroxybenzoic acid require 50 and 160 μg/mL, respectively [11, 12]. Similarly, hydroxylated aromatic compounds exhibit more potent free radical scavenging activity and lower cytotoxicity. The addition of caffeic acid increases superoxidase dismutase activity by 3050 folds and reduces cytotoxicity by five times when compared to 4-coumaric acid [13]. Furthermore, 3,4,5-trihydroxycinamic acid exhibits 4 folds higher suppression of lipopolysaccharide-induced NO production compared to caffeic acid [14].

**Fig. 1:**
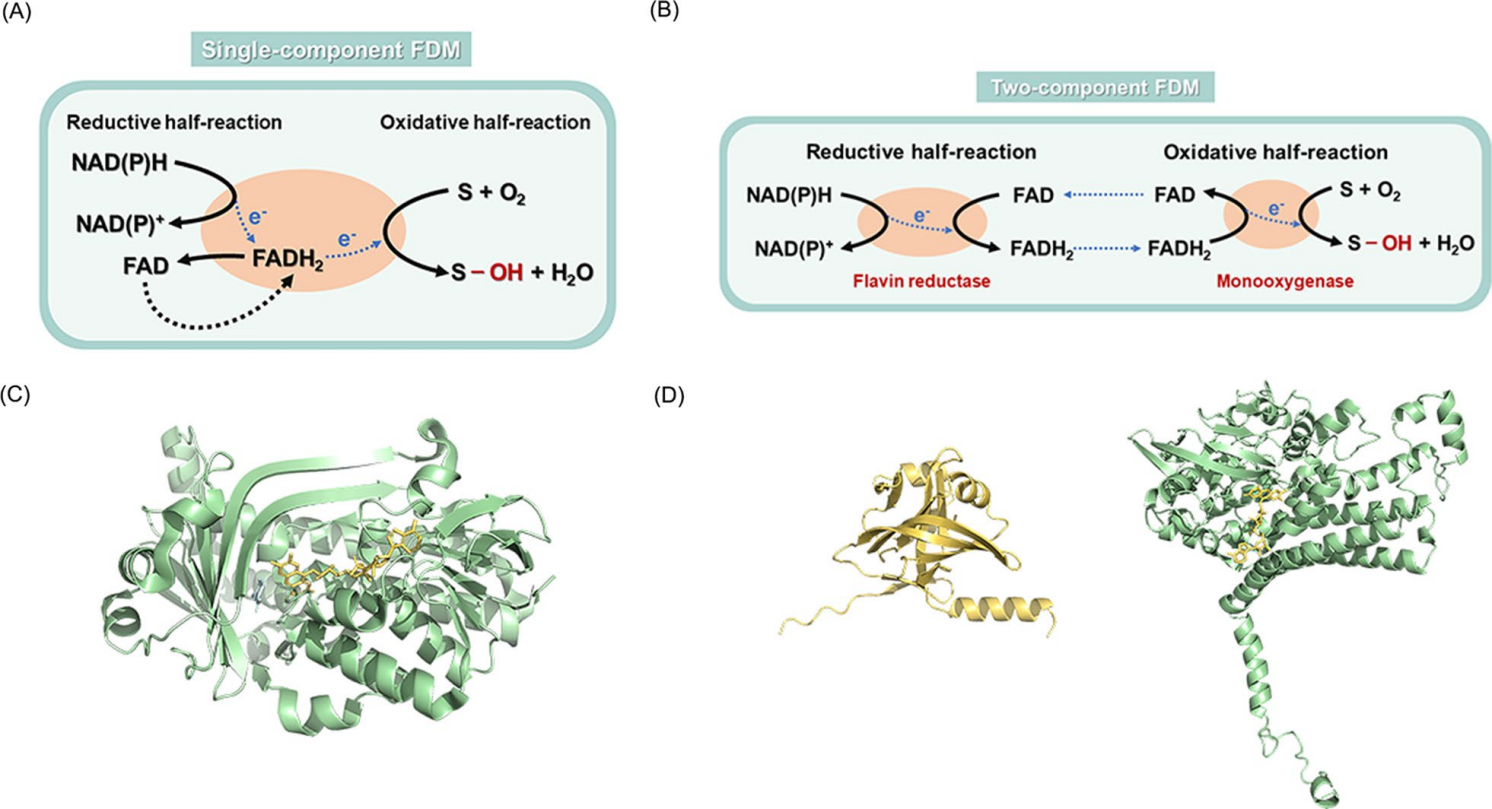
Catalytic mechanism of single- and two-component FDMs. S: substrate. **A** In single-component flavin-dependent monooxygenases (FDM), NAD(P)H serves as the reducing power to reduce the flavin cofactor FAD after binding the substrate, resulting in the formation of a reduced complex in the reductive half-reaction. Subsequently, the reduced complex reacts with molecular oxygen to hydroxylate the substrate and regenerate the oxidative flavin cofactor FAD in the oxidative half-reaction. **B** In two-component FDMs, FAD is bound with flavin reductase as the flavin cofactors and is reduced directly by NAD (P)H without the need to bind the substrate. Once reduced, the flavin cofactor FADH_2_ is released and transferred to the monooxygenase component. The subsequent catalytic steps of the monooxygenase are similar to the oxidative half-reaction of single-component FDMs, where FADH_2_ is used to hydroxylate the substrate. **C** Protein structure of a typical single-component FDM: PobA from *Pseudomonas aeruginosa* [50]. **D** Protein structures of typical two-component FDM: *Ec*HpaB (right) and *Ec*HpaC (left) from *Escherichia coli* [63, 99]

Hydroxylated aromatic compounds are obtained by catalytic reactions of hydroxylases. Three primary types of hydroxylases are widely reported, cytochrome P450 (CYP450), pterin-dependent monooxygenase (PDM), and flavin-dependent monooxygenase (FDM). CYP450, a family of heme-containing monooxygenases, is known for its versatility in performing a diverse range of oxidative reactions, and comprehensive reviews on this topic are readily available [15–19]. PDM relies on specific pterin cofactors, tetrahydrobiopterin (BH4) and tetrahydromonapterin (MH4), which are essential for catalytic reactions but are often found in limited intracellular quantities [20–23]. And most PDM is primarily used for hydroxylating aromatic amino acids, its potential applications are still under exploration and won't be discussed in depth here [24]. FDM, a superfamily of enzymes with flavin as a cofactor, functions as monooxygenase [25]. FDM can be classified into eight groups, designated as A to H. Groups A and B are single-component enzymes that depend on NAD(P)H as external electron donor to supply reducing force. Groups C to F are two-component systems, which consist of a monooxygenase and a flavin reductase. Groups G and H are also single-component with the ability to reduce the flavin cofactor by oxidating substrates [26, 27]. The catalytic mechanism of FDM has been fully reported in detail [28–32]. In brief, the FDM-catalyzed hydroxylation of aromatic compounds can be divided into two parts: the reductive half-reaction for FAD and FMN, and the oxidative half-reaction for substrates [33]. As shown in Fig. 1, single-component FDM can catalyze both half-reactions at the same active site, while two-component FDM requires two separate enzymes, flavin reductase and monooxygenase, to participate in each half-reaction [34, 35]. Because of its small molecular weight, free expression without membrane anchor, and relatively high cofactor content, FDM is the preferred choice for the production of hydroxylated aromatic compounds when compared to the previous two types of enzymes [36–40].

While FDMs have been employed to establish biosynthetic pathways for producing hydroxylated aromatic compounds, its catalytic efficiency remains insufficient for large-scale industrial production [41, 42]. Consequently, numerous studies have been dedicated to enhancing the catalytic efficiency of FDMs. In this paper, we present a summary of three strategies aimed at enhancing catalytic efficiency of FDMs: (a) development of efficient enzyme mutants through protein engineering; (b) enhancement and acceleration of cofactor supplementation and recirculation; (c) facilitating cofactors delivery for enhancing FDMs catalytic efficiency. These strategies have been investigated and applied, resulting in a significant improvement in FDMs' hydroxylating efficiency enhancing. We also discuss the prospects of protein scaffold and computational enzyme design strategy as promising avenues for enhancing the catalytic efficiency of FDMs.

## Strategies to improve the catalytic activity of FDM

### Developing efficient FDM mutants by protein engineering

Protein engineering has progressed through different stages over the past 50 years, including primary rational design, directed evolution, semi-rational or rational design, and computational design [43]. Through these stages, protein engineering has facilitated the acquisition of new properties by enzymes, such as broadening their substrate range and enhancing catalytic efficiency [44]. Recently, protein engineering has proven its effectiveness and potential promise in obtaining efficient mutants for FDMs.

**Fig. 2:**
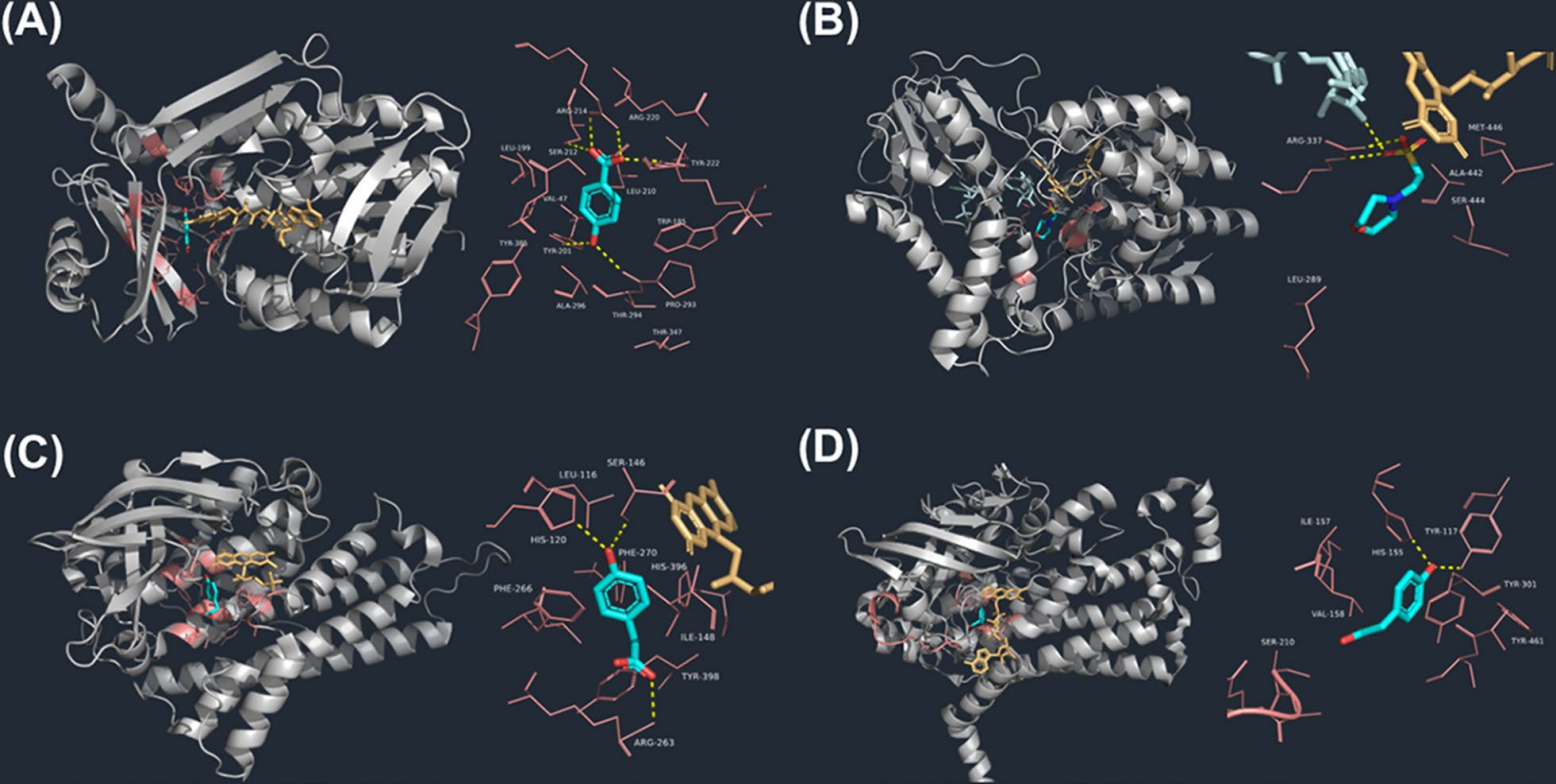
Structures of FDM-substrate complexes and their active center. Gray: Protein structure; Salmon: Animo acid residues of active center; Lightorange: Flavin cofactor; Cyan: Substrate. **A** Structure of PobA (PDB ID: 1IUV) and 4-HBA; **B** Structure of *Tf*BVMO from *Thermobifida fusca* (PDB ID: 2YLT) and 2-(N-morpholino)-ethanesulfonic acid; **C** Structure of *Ab*HpaB from *Acinetobacter baumannii* (PDB ID: 2JBT) and 4-HPA; **D** Structure of *Ec*HpaB from *Escherichia coli* (PDB ID: 6QYI) and 4-HPA

The wild-type PobA enzyme can convert 4-hydroxybenzoic acid (4-HBA) into 3,4-dihydroxybenzoic acid (3,4-DHBA), but lacks the capability to further hydroxylate 3,4-DHBA into the more valuable 3,4,5-trihydroxybenzoic acid (Gallic acid, GA). Analysis of the co-crystallization of PobA with 4-HBA revealed that Y201 and Y385 in the catalytic site of PobA form a hydrogen bonding network with 4-OH group of 4-HBA **(**Fig. 2**)**. Specifically, Y201 activates 4-HBA, and the product 3,4-DHBA is released at the end of the catalytic reaction [45]. Notably, after mutating Y385 to phenylalanine, the hydrogen bonding network was destroyed, and the flexibility of the catalytic site was increased. This change allows 3,4-DHBA to be rotated 180° around the C1-C4 axis in the site and be secondarily hydroxylated to produce new product GA [46, 47]. Further insight was gained through the co-crystallization of 3,4-DHBA with PobA, revealing that the 4-OH group of 3,4-DHBA forms hydrogen bonds with amino acid residues T294, P293, Y201, and T347 [48]. Mutating T294 to alanine resulted in the disruption of the hydrogen bond connecting T347 and this mutation also led to a reduction in steric hindrance of the substrate at the catalytic site. Compared to mutant Y385F, the catalytic activity of the PobA mutant Y385F/T294A towards 3,4-DHBA increased by 60 folds, the value of *K*_M_ decreased to 157.02 ± 31.41 μM and *k*_cat_ raised to 1.69 ± 0.18 s^−1^. Furthermore, its activity towards 4-HBA increased by 2 folds [48] (Table 1). Moreover, the mutation of L199 to valine significantly decreased uncoupling reaction, enabling the mutant to better immobilize the C4a-hydroperoxy flavin intermediate and improve its utilization in substrate hydroxylation [49]. The PobA mutant Y385F/L199V displayed a 425-fold increase in catalytic activity for 3,4-DHBA compared to the Y385F mutant, with a decrease in *K*_M_ to 52 ± 16 μM and increase in *k*_cat_ to 4.4 ± 0.4 s^−1^, meanwhile its catalytic activity for 4-HBA was also increased by 2 folds [50] (Table 1).

**Table 1:** Kinetic parameters of PobA mutants in different studies

Variants	4-HBA			3,4-DHBA			References
	*K*_*M*_	*k*_*cat*_	*k*_*cat*_/*K*_*M*_	*K*_*M*_	*k*_*cat*_	*k*_*cat*_/*K*_*M*_	
	(μM)	(s-^1^)	(μM^−1^ s-^1^)	(μM)	(s-^1^)	(μM^−1^ s-^1^)	
Wide type	34.67 ± 9.51	14.12 ± 1.49	0.41	–	–	–	[47]
Y385F	19.55 ± 0.32	0.20 ± 0.001	0.01	228.02 ± 53.57	0.39 ± 90.06	0.0002	
Y385F/T294A	48.38 ± 3.00	0.90 ± 0.03	0.02	157.02 ± 31.41	1.69 ± 0.18	0.012	
Y385F/L199V	22 ± 5	0.45 ± 0.02	0.021	52 ± 16	4.4 ± 0.4	0.085	[49]
L199R/T294C/Y385M	24 ± 10	1.9 ± 0.2	0.079	48 ± 10	3.0 ± 0.2	0.062	[56]
V47I/L199N/T294A/Y385I	120 ± 10	4.0 ± 0.1	0.034	120 ± 10	4.1 ± 0.2	0.033	
Y385F/T294A/V349A	14.5 ± 2.5	1.36 ± 0.04	0.094	30.3 ± 10.4	1.78 ± 0.16	0.059	[57]

Additionally, the substrate spectrum of Baeyer–Villiger Monooxygenases (BVMO) was expanded by protein engineering [51, 52]. The BVMO mutant M466G from *Thermobifida fusca* was capable of converting indole to indigo, a high-value industrial dye, whereas the wild type lacked this catalytic ability **(**Fig. 2**)** [53]. This is caused by the shortening of the amino acid side chain around the active site, which further enlarges the catalytic space. Besides, the R292 of BVMO from *Acinetobacter radioresistens* S13 was mutated to test its effect on the enzymatic specificity [54]. The results showed that the mutant R292A efficiently hydroxylated indole into both indigo and indirubin, whereas the wild type lacked this capability [55]. Under optimal conditions for whole-cell catalysis, the mutant was able to yield 154 mg/L indigo and 138 mg/L indirubin [56].

These studies indicate the potential benefits of modifying the hydrogen bonds between the substrates and the catalytic site to enhance the flexibility during mutant enzyme design. The elimination of hydrogen bond effects allows substrates to move more freely within the catalytic site, resulting in more efficient catalytic reactions.

To streamline the process of obtaining efficient mutants, several high-throughput screening methods have been established based on the substrate, product chromogenic reaction, and cofactor supplement within the PobA-catalyzed reaction. An innovative screening platform based on the *Escherichia coli* (*E. coli*) growth stage has been developed to couple the catalytic efficiency of the enzyme with the state of cell growth, making the results of high-throughput screening more intuitive. In this screening platform, the key genes responsible for NADP^+^ regeneration in the platform strain are knocked out, and NADP^+^ regeneration is closely linked to the hydroxylase catalytic reaction. The hydroxylating reactions convert NADPH to NADP^+^, which is crucial for maintaining the redox balance of NADPH in the strain and is also essential for cell growth. Consequently, the enhanced catalytic efficiency of the hydroxylase leads to more effective NADP^+^ replenishment and a better growth state of the strain [57]. Utilizing this method, the PobA triple mutant L199R/T294C/Y385M was screened out, which exhibited 310 folds increase in activity towards 3,4-DHBA, and a 7.9 folds increase in activity towards 4-HBA compared to mutant Y385F. The *K*_M_ value decreased to 48 ± 10 μM and *k*_cat_ raised to 3.0 ± 0.2 s^−1^ for 3,4-DHBA, while the *K*_M_ value increased to 24 ± 10 μM and *k*_cat_ raised to 1.9 ± 0.2 s^−1^ for 4-HBA (Table 1). Moreover, another PobA triple mutant Y385F/T294A/V349A was screened out using the chromogenic reaction of GA with 0.1 M NaHCO_3_ [58]. This mutant showed a 295 folds increase in activity towards 3,4-DHBA and 9.4 folds enhancement catalytic activity to the 4-HBA in comparison to Y385F mutant. The *K*_M_ value decreased to 30.3 ± 10.4 μM and *k*_cat_ raised to 1.78 ± 0.16 s^−1^ for 3,4-DHBA, meanwhile the *K*_M_ value decreased to 14.5 ± 2.5 μM and *k*_cat_ raised to 1.36 ± 0.04 s^−1^ for 4-HBA (Table 1).

HpaBC is a typical class of two-component flavin monooxygenase, consisting of the monooxygenase HpaB and the flavin reductase HpaC. Recent researches have focused on mutating HpaB from different origins to generate improved mutant enzymes. The wild-type *Ab*HpaB from *Acinetobacter baumannii* can effectively catalyze 4-hydroxyphenylacetate (4-HPA) to 3,4,5-trihydroxyphenylacetate, but it is inefficient to catalyze 4-coumaric acid to 3,4,5-trihydroxycinnamic acid (3,4,5-THCA) (Fig. 2) [59]. To enhance its catalytic capabilities, site-directed mutations were performed on the residues located within 4 Å of the catalytic site, including I148, R263, and Y398. These residues were modified into smaller ones to better accommodate 4-coumaric acid, which has a longer carboxylic acid tail compared to 4-HPA. The outcome was the Y398S mutant, which displayed an increased conversion efficiency of 4-coumaric acid to 3,4,5-THCA, with conversion rates rising from 26 to 50% [60, 61] (Table 2). In another study, the R263E mutant demonstrated the ability to catalyze the *o*-hydroxylation of tyramine, R263D/Y398D double mutant effectively catalyzed the conversion of octopamine to norepinephrine, while the wild type lacked any activity toward either tyramine or octopamine [62]. Furthermore, the crystal structure of *Ec*HpaB from *E. coli* was elucidated, revealing a unique loop structure that covers the catalytic sites. This loop was found to be remarkably flexible and amenable to extensive mutations, allowing for substrate access and stable binding to the catalytic sites (Fig. 2) [63]. Thus, mutations in this loop hold the promise of yielding valuable enzyme mutants. Specifically, residues S210, A211, and Q212 in this loop were selected for saturated mutation, the resulting mutant enzyme, 23F9-M4, exhibited a remarkable 95% increase in the conversion rate to tyrosine compared to the wild-type enzyme, but displaying significantly reduced activity towards the natural substrate, 4-HPA [64] (Table 2). When this loop was designed and generated as a random coil, the *K*_*M*_ values for non-natural larger substrates such as resveratrol and naringenin decreased from 174.3 μM, 349.8 μM, to 144.0 μM, 191.6 μM, respectively. Additionally, the mutants retained almost the same specificity constant values (*k*_cat_/*K*_*M*_) for small substrates like 4-coumaric acid and umbelliferone. This suggests that the loop can be effectively engineered to accommodate larger substrates while maintaining specificity for smaller ones. A structural comparison between *Ro*HpaB from *Rhodococcus opacus* which obtained through homology modeling and *Ec*HpaB, revealed that the loop region of *Ro*HpaB has a shorter chain length and a smaller substrate-binding pocket [65]. This structural difference makes it challenging for large aromatic compounds to enter the pocket. To address this, the *Ro*HpaB mutant Y215A was generated through alanine scanning of amino acid residues 212 to 222 in the loop region. This mutant exhibited a remarkable 25.3 folds increase in catalytic activity towards naringenin, with a *K*_M_ value of 3.0 μM and a *k*_cat_ value of 0.02 s^−1^ [65]. The improved catalytic performance of mutant Y215A can be attributed to the smaller side chain of alanine resulted in a larger substrate-binding pocket, allowing the substrate to enter and exit the catalytic pocket more readily. Furthermore, this mutant also demonstrated catalytic activities towards apigenin and kaempferol, which were not observed in the wild type enzyme. This showcases the potential for engineering the loop region to enhance the catalytic versatility of HpaB. Consequently, mutating the amino acid residues around the catalytic site to smaller residues can enlarge the space for accommodating substrates, which increases the catalytic rate and broadens the substrate spectrum.

**Table 2:** Kinetic parameters of HpaB mutants in different studies

Enzymes	Variant	Substrates	Products	Convertion rate (%)	References
AbHpaB	Wild type	Tyramine	Dopamine	ND	[59]
	R293A			7	
	R263D			57	
	Wild type	4-Coumaric acid	3,4,5-THCA	7.5	[60]
	Y398S			23.2	
	Wild type	Octopamine	Norepinephrine	ND	[61]
	R263D/Y398D			13	
EcHpaBC	Wild type	Tyrosine	L-Dopa	1.01	[63]
	23F9-M4			95.5	
EcHpaBC	Wild type	Tyrosol	Hydroxytyrosol	4.87	[65]
		Tyramine	Dopamine	0.1	
	S210T/A211L/Q212E	Tyrosol	Hydroxytyrosol	95.2	
		Tyramine	Dopamine	2.15	
	A211G / Q212Y	Tyrosol	Hydroxytyrosol	7.23	
		Tyramine	Dopamine	90.54	
	S210T/A211M/Q212G	Tyrosol	Hydroxytyrosol	97.33	
		Tyramine	Dopamine	45.51	

A high-throughput screening method using chromogenic reactions of products and sodium periodate was also employed to screen efficient mutants of *Ec*HpaB capable of catalyzing tyrosine or tyramine. Among the mutants, the mutant S210T/A211L/Q212E exhibited a catalytic activity on tyrosine that was 19 folds higher than the wild type, and the mutant A211G/Q212Y demonstrated a remarkable 386-fold increase in catalytic activity for tyramine compared to the wild type. Additionally, the mutant S210T/A211M/Q212G displayed enhanced conversion rate towards tyrosol and tyramine, with increasing by 17 and 271 folds, respectively [66] (Table 2).

The introduction of single-point mutations or combinatorial mutations in the amino acids surrounding FDMs' catalytic site enables the alteration of the catalytic pocket's size and its interactions with substrates, results in improved catalytic efficiency and broadens the substrate spectrum for FDMs. Furthermore, the high-throughput screening of FDM mutants has the potential to yield unexpectedly positive mutants, even when the mutation sites are seemingly distant from the catalytic site. In summary, with the assistance of protein engineering, a diverse range of FDM mutants has been developed and successfully applied in the biosynthesis of hydroxylated aromatics. This presents new possibilities for the extensive utilization of FDMs in various applications.

### Enhancing FDM catalytic effiency by cofactor engineering

FDMs are reliant on cofactors, which also be regarded as co-substrates, consuming flavin cofactors FADH_2_/FMNH_2_ and nicotinamide cofactors NAD(P)H/NAD(P)^+^ during catalytic reactions. Insufficient cofactors supplementation can hinder the efficiency of hydroxylation reactions [67]. To address this issue, the most economically feasible approach involves the efficient recirculation of cofactors within production systems, as long-term cofactor storage is both costly and challenging [68, 69]. To tackle the problem of inadequate cofactor supply, it has been demonstrated that increasing cofactor production, accelerating cofactor regeneration, and minimizing cofactor consumption by other metabolic pathways can effectively improve hydroxylation efficiency.

**Fig. 3:**
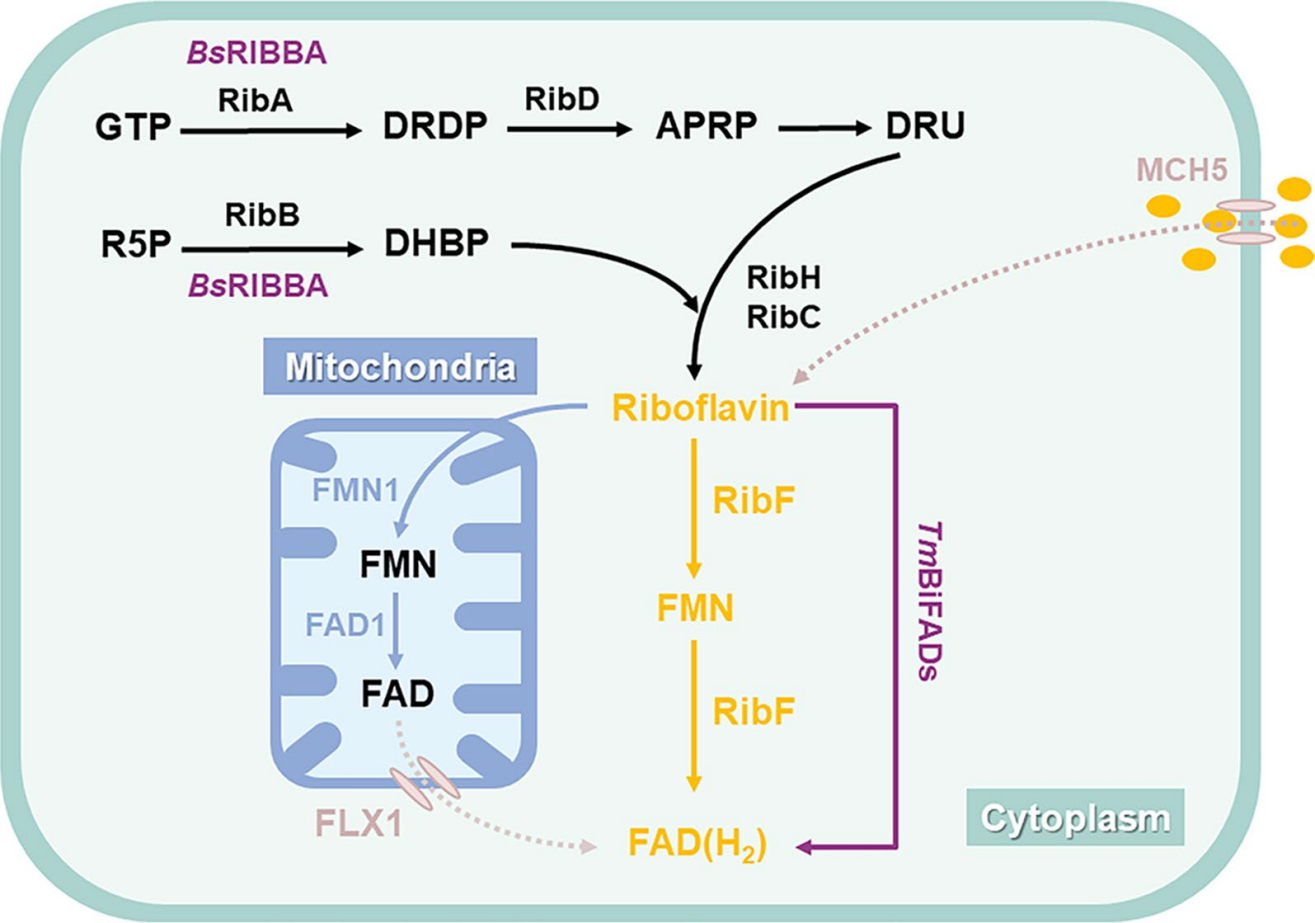
Biosynthesis and recirculation of FADH_2_. Guanosine triphosphate (GTP), GTP cyclohydrolase II (RibA), 2,5-Diamino-6-(5-phospho-D-ribosylamino) pyrimidin (DRDP), 5-amino-6-(5-phospho-ribosylamino) uracil reductase (RibD), 5-Amino-2,6-dioxy-4-(5ʹ-phospho-D-ribitylamino) pyrimidine (APRP), 5-Amino-6-(D-ribitylamino) uracil (DRU), Ribulose 5-phosphate (R5P), DHBP synthase (RibB), L-3,4-Dihydroxybutan-2-one 4-phosphate (DHBP), 6,7-dimethyl-8-ribityllumazine synthase (RibH), riboflavin synthase (RibE), DHBP synthase/GTP cyclohydrolase II (BsRIBBA), riboflavin kinase/FAD synthase (TmBIFADs), riboflavin kinase (FMN1, RibF), FAD synthetase (FAD1)

FADH_2_/FMNH_2_ serves as the essential cofactor for FDM. It directly contacts the substrate and delivers reactive oxygen atoms to the substrate to form hydroxyl groups. Consequently, the availability of this cofactor significantly influences the catalytic activity of FDM. [41]. As shown in Fig. 3, initiating from GTP and R5P, the biosynthesis of FADH_2_/FMNH_2_ engages six enzymes (RibA*,* RibB, RibC, RibD, RibF, RibH) [70]. Among these enzymes, RibA and RibB, as the first step enzymes in the FADH_2_ biosynthetic pathway, are used to convert GTP to DRDP and R5P to DHPB, respectively, but they are both allosterically inhibited by FADH_2_ [71]. RibC, RibF, and RibH are identified as primary rate-limiting enzymes in the FADH_2_ biosynthetic pathway [72]. In prokaryotes, riboflavin is converted to FAD by RibF in the cytoplasm, whereas in eukaryotes, riboflavin enters the mitochondria and is converted to FAD by FMN1 and FAD1. Finally, FAD is transported out into the cytoplasm by the transport protein FLX1 [73] **(**Fig. 3**)**.

Overexpressing enzymes in the FADH_2_ biosynthetic pathway is a conventional and effective method to enhance FADH_2_ concentration. In yeast, the overexpression of RibA and FLX1 resulted in a 51.7% increase in caffeic acid production, reaching 244 mg/L, primarily attributed to increased FDM efficiency facilitated by increasing availability of FADH_2_ **(**Fig. 3**)** [74, 75]. Moreover, to address the issue of endogenous enzyme inhibition caused by pathway products, the introduction of efficient heterologous isozymes has proven effective. To mitigate the allosteric inhibition of endogenous enzymes by FADH_2_, the bifunctional *Bs*RIBBA from *Bacillus subtilis* was chosen as a substitute for RibA and RibB. Meanwhile, *Tm*BiFADS from *Thermotoga maritima* was selected as a substitute for RibF, as its higher catalytic efficiency. Overexpression of *Bs*RIBBA and *Tm*BiFADS further increased the supply of FADH_2_ to FDM (Fig. 3), resulting in a 54% boost in caffeic acid production, reaching 248.2 mg/L [74, 76]. In addition, this study also overexpressed the endocytotic protein MCH5 **(**Fig. 3**)**, which was able to absorb riboflavin from the culture medium and accumulate it intracellularly. When MCH5 was overexpressed, caffeic acid production was increased by 97%, reaching 311 mg/L [74].

Accelerating the intracellular recirculation of FADH_2_ is indeed a beneficial strategy to ensure a self-sufficient supply of intracellular cofactors and minimize the burden on cell growth. Achieving this self-sufficiency in intracellular cofactors is crucial to support the catalytic reactions of FDMs effectively. Recirculation of FADH_2_ requires the active participation of flavin reductase and NAD(P)H. By introducing flavin reductases into biosynthetic pathways, it's possible to facilitate the reduction of FAD to FADH_2_ using NAD(P)H as the reducing agent. This introduction of flavin reductase enhances the catalytic activity of FDMs by expediting the recirculation of FADH_2_. However, it's essential to be mindful that this improvement in FDMs activity comes at the expense of increased NAD(P)H consumption. NAD(P)H is not only a crucial cofactor for enzyme catalysis but also a vital component for cellular growth and metabolism. An imbalance in the NAD(P)H/NAD(P)^+^ ratio can adversely affect overall cellular health and functionality, as it impacts both enzyme-catalyzed reactions and broader cellular processes. Therefore, when implementing strategies to accelerate cofactor recirculation, it is important to carefully manage the NAD(P)H balance to ensure the optimal functioning of the entire cellular system [77].

**Fig. 4:**
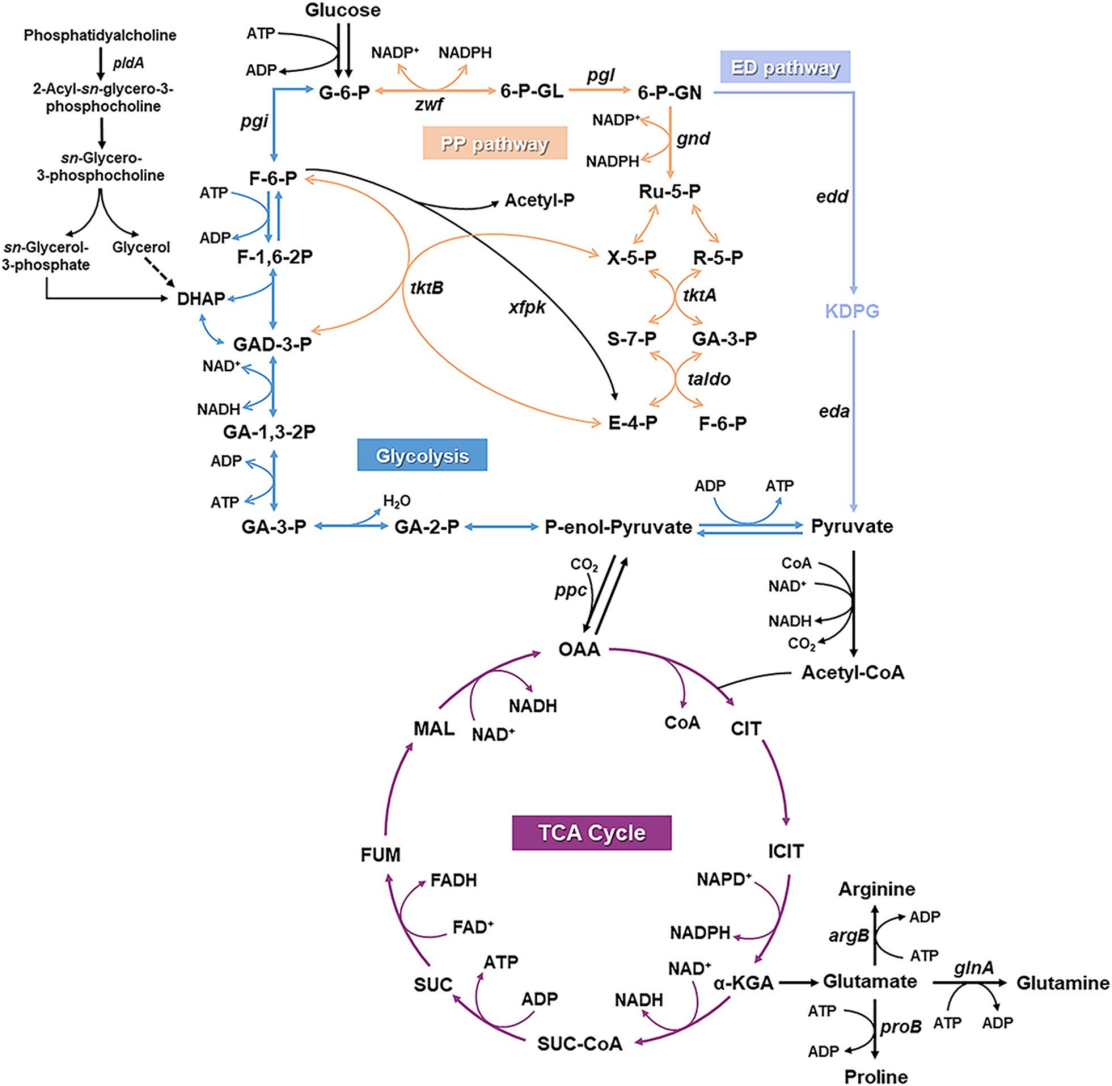
The intracellular biosynthetic network of NAD(P)H. Glucose-6-phosphate (6-G-P), glucose-6-phosphate isomerase (*pgi*), fructose-6-phosphate (F-6-P), fructose 1,6-bisphosphate (F-1,6-2P), dihydroxyacetone phosphate (DHAP), glyceraldehyde 3-phosphate (GAD-3-P), glycerate 1,3-diphosphate (GA-1,3-2P), glycerate 3-phosphate (GA-3-P), glycerate 2-phosphate (GA-2-P), phosphoenolpyruvate (P-enol-Pyruvate), phosphoenolpyruvate carboxylase (*ppc*), glucose-6-phosphate 1-dehydrogenase (*zwf*), 6-phospho-D-glucono-1,5-lactone (6-P-GL), 6-phosphogluconolactonase (*pgl*), 6-phospho-D-gluconate (6-P-GN), 6-phosphogluconate dehydrogenase (gnd), ribulose 5-phosphate (Ru-5-P), xylulose 5-phosphate (X-5-P), ribose 5-phosphate (R-5-P), transketolase (*tktA,tktB*), sedoheptulose 7-phosphate (S-7-P), transaldolase (*taldo*), erythrose 4-phosphate (E-4-P), Phosphoketolases (*xfpk*), phosphogluconate dehydratase (*edd*), 2-Keto-3-deoxy-6-phosphogluconate (KDPG), 2-dehydro-3-deoxyphosphogluconate aldolase (*eda*), oxaloacetate (OAA), citrate (CIT), isocitrate (ICIT), *α*-ketoglutaric acid (*α*-KGA), succinyl-CoA (SUC-CoA), succinate (SUC), fumarate (FUM), malate (MAL), acetylglutamate kinase (*argB*), glutamate 5-kinase (*proB*), glutamine synthetase (*glnA*), phospholipase A (*pldA*)

In cells, NADPH is primarily produced by the oxidative branch of the pentose phosphate pathway (PP pathway). As shown in Fig. 4, the enzyme G6PDH (encoded by *zwf*) converts glucose-6-phosphate (G-6-P) to 6-phosphoglucono-δ-lactone (6-P-GL) and reduces NADP^+^ to NADPH. Then, 6PGDH (encoded by *gnd*) converts 6-phosphogluconate (6-P-GN) to ribulose-5-phosphate (Ru-5-P) and reduces NADP^+^ to NADPH. NADH is mainly generated through the glycolysis and the tricarboxylic acid cycle (TCA cycle) [78, 79]. Besides, 6-P-GN generated by PP pathway can be rapidly catalyzed to produce pyruvate through the Entner-Doudoroff pathway (ED pathway). This pyruvate is then converted into acetyl coenzyme A (Acetyl-CoA), which participates in the TCA cycle while generating substantial amounts of NADH [80].

To enhance the supply of NADPH, one effective approach is redirecting metabolic flux into the PP pathway [81, 82]. Overexpression of G6PDH resulted in a 1.5 folds increase in the intracellular NAD(P)H concentration, elevating it from 200 nmol/g cell to 297 nmol/g cell [83]. This increase in NAD(P)H concentration led to a substantial 1.56 folds enhancement in the production of hydroxyl-epoxyprogesterone, reaching 14.6 g/L [84]. Overexpression of genes, *xfpk*, *pta*, *taldo*, *tktA* **(**Fig. 4**)**, can successfully divert more metabolic flux into the PP pathway, leading to increased NADPH production. This adjustment significantly bolsters the availability of cofactors required for FDM catalysis. In the engineered strain, the NADPH/NADP^+^ ratio was 46% higher compared to the control strain. As a result, the production of caffeic acid increased from 157 mg/L to 385 mg/L [74]. These results identified the effectiveness of overexpressing genes of PP pathway in promoting cofactor availability and enhancing the catalytic efficiency of FDMs in biosynthetic processes.

Blocking metabolic flux to competing pathways is another viable approach to enhance the supply of NAD(P)H. For example, in the biosynthesis of catechin, a compound known for its various antioxidant and antitumor properties, strategies include the knockout of the *pgi* gene to prevent G6P from entering glycolysis, the knockout of *ppc* to redirect metabolic flux towards the TCA cycle, and the knockout of *pldA* (encoding phospholipase A) to reduce GAD-3-P consumption were used to supply more NAD(P)H. After implementing these strategies, the production of catechin remarkably increased from 39 mg/L to 374 mg/L [85]. TCA cycle is a significant source of NADH, but it also contains metabolic bypasses that divert metabolites away from generating reducing cofactors. By inhibiting the expression of genes like *argB* (encoding acetylglutamate kinase), *proB* (encoding glutamate 5-kinase), and *glnA* (encoding glutamine synthetase) using CRISPRi **(**Fig. 4**)**, it's possible to block the generation of arginine, proline, and glutamate from α-KGA. This inhibition results in an increase in intracellular NADH content by 1.3 folds, ultimately leading to an enhancement in pinocembrin production from 14.3 mg/L to 39.8 mg/L. This approach effectively redirects metabolic flux toward the generation of NADH. These highlight the effectiveness of channeling metabolic flux into NAD(P)H-producing pathways, which significantly enhances the hydroxylation efficiency of the hydroxylated aromatic compounds biosynthetic pathway. In addition, the ED pathway, serving as an alternative to glycolysis, has the remarkable capability to rapidly generate pyruvate through a concise 4-step reaction **(**Fig. 4**)**. This allows for the efficient utilization of metabolic flux to produce pyruvate and acetyl-CoA, which can readily participate in the TCA cycle, yielding large amounts of NADH. A modular plug-in ED pathway was developed to efficiently generate NAD(P)H. This module incorporates five genes *pgi,zwf*, *pgl*, *edd* and *eda* from *Zymomonas mobilis* [86] **(**Fig. 4**)**. By co-expressing these genes under different promoters and operators, various ED plasmid modules were created. When these modules were introduced into *E. coli* strains separately, the strain with the plasmid ED11 exhibited a remarkable 25 folds increase in NADPH production efficiency compared to the wild-type strain [86]. Applying this module to produce vanillin, the hydroxylation efficiency of FDM was enhanced by 1.97 folds through rapid NADPH generation, resulting in a 17 folds boost in vanillin production to 585 mg/L [87]. This modular approach to enhancing cofactor supply for FDM holds great promise and is an efficient strategy for improving its catalytic performance.

In conclusion, by driving metabolic flux to the NAD(P)H-producing pathway is a very efficient way to obtain massive amounts of cofactors. Overexpression of either the oxidative or non-oxidative pathway enzymes of the PP pathway could increase the supply of NADPH, thereby improving the catalytic efficiency of FDM. Moreover, by blocking metabolic flow driving toward pathways that do not produce cofactors, more metabolic flow is conserved to regenerate cofactors. In addition, rapidly generating metabolic flux to cofactor through the ED pathway is a novel and promising strategy to enhance the supply of cofactors for FDM catalysis.

### Facilitating cofactors delivery for enhancing FDM catalytic efficiency

During catalytic process, FDM uses FADH_2_ as a cofactor to hydroxylate substrates. The mechanism for cofactor utilization differs between single-component FDM and two-component FDM. In single-component FDMs, the enzyme can directly utilize NAD(P)H to reduce FAD to FADH_2_ within the enzyme itself. This allows the enzyme to carry out the hydroxylation of substrates using the cofactors generated within its own structure. In contrast, two-component FDM, specifically the monooxygenase component, require a flavin reductase to facilitate the conversion of FAD to FADH_2_. This process consumes NAD(P)H. Once FADH_2_ is generated by the flavin reductase, it is then delivered to the monooxygenase, where it participates in the oxidation of substrates [88]. Thus, the efficiency of cofactor delivery between the monooxygenase and flavin reductase directly impacts the catalytic efficiency of two-component FDM. Effective and timely delivery of FADH_2_ to the monooxygenase ensures that the enzyme has a sufficient supply of cofactors to efficiently carry out substrate hydroxylation.

To optimize the catalytic efficiency of two-component FDM, combining monooxygenase and flavin reductase components from different sources can be a valuable strategy. For example, in a study aimed at producing caffeic acid in yeast, researchers employed monooxygenase (HpaB) and flavin reductase (HpaC) components from four different microbial origins [89]. When they expressed homologous HpaB and HpaC from *Pseudomonas aeruginosa*, the highest production of caffeic acid achieved was 68.2 mg/L. However, by combining components from different origins, specifically using PaHpaB from *P. aeruginosa* and PlHpaC from *Photorhabdus luminescens*, they achieved the highest caffeic acid production of 241.3 mg/L [89]. Similarly, the combination of monooxygenase SgcC from *Streptomyces globisporus* with the different flavin reductase SgcE6 (from *S. globisporus*), Fre (from *E.coli*), VlmR (from *Streptomyces viridifaciens*), RebF (from *Streptomyces coelicolor*), led to varying degrees of improvement in reducing FAD by 3 folds, 2 folds, 13 folds, 5 folds, respectively [90]. These studies demonstrate that selecting an optimal combination of monooxygenase and flavin reductase from various sources can significantly enhance the catalytic efficiency of two-component FDMs for specific applications. In addition to combining monooxygenase and flavin reductase components from different microbial origins, the generation of HpaC mutants offers another approach to optimize the efficiency of two-component FDMs. In a study involving HpaB and HpaC from *Acinetobacter baumannii* [91], a variety of HpaC mutants were obtained through targeted mutagenesis then co-expressed with HpaB to test their catalytic capabilities. When the HpaC A58P was co-expressed with HpaB, it exhibited a 1.82 folds increase in the conversion rate of caffeic acid to 3,4,5-THCA, rising from 8.5 to 15.5 μM/min. This improvement can be attributed to the enhanced thermostability of the HpaC mutant, which is 5.6 times more stable, 3.5 times more efficient in reducing FAD, and capable of providing FADH_2_ for a longer duration during the catalytic reaction [91].

Reducing the spatial distance between the two components of FDMs, specifically monooxygenase and flavin reductase, can enhance cofactor delivery and improve the catalytic efficiency. One approach to achieve this is through fusion expression, where the C-terminus of one component is connected to the N-terminus of the other using linkers. For instance, a flexible chain polypeptide GGGGS was used to connect C-termini of HpaB and N-termini of HpaC. When this fusion protein was introduced with other pathway genes into *C. glycerinogenes* for the de novo biosynthesis of caffeic acid, the production of caffeic acid was increased to 21.9 mg/L, a 1.48 folds enhance compared to separate expression [92]. Furthermore, linking flavin reductase (Fre) to the N-terminus of HpaB using two different types of linkers, a flexible linker (FL) and a rigid linker (RL), resulted in the formation of fusion proteins Fre-FL-HpaB and Fre-RL-HpaB. This fusion approach led to remarkable increases in caffeic acid production, with a 7.5 folds increase to 748.2 mg/L for Fre-FL-HpaB and a 9.1 folds increase to 907.1 mg/L for Fre-RL-HpaB in *E. coli* [93]. The improved production of caffeic acid was primarily attributed to the close spatial proximity between Fre and HpaB, enabling Fre to efficiently provide FADH_2_ for the catalytic reaction of HpaB. This innovative method highlights the significance of reducing the spatial distance between key components in FDMs to boost catalytic efficiency.

In conclusion, the hydroxylated efficiency of FDM can be significantly improved by co-overexpressing monooxygenases and flavin reductases through combinatorial screening and linker application. This improvement could be due to the increase of reduced flavin production rate. However, this approach is not always beneficial, particularly in whole-cell biocatalysts because some flavin reductases are ineffective at immobilizing the C4a-hydroperoxy flavin intermediate, and uncoupling reactions waste the reactive oxygen carried by cofactors, resulting in the consumption of NAD(P)H and the formation of cell-damaging H_2_O_2_ [94, 95]. Therefore, further exploration is required to identify more efficient flavin reductases or to mutate existing reductases to decrease uncoupling reactions.

## Conclusions

Enhancing the hydroxylating efficiency of various FDMs in the biosynthetic pathway of aromatic compounds has been effectively achieved through protein engineering, cofactor engineering, and facilitating cofactor delivery. However, several challenges still need to be addressed.

Firstly, a significant challenge is the identification of suitable mutant sites in FDMs. The integration of advanced computational methods like AlphaFold2 for predicting protein structures and molecular dynamics simulations for understanding protein dynamics and interactions is indeed promising. These methods have the potential to provide valuable insights into FDMs' catalytic sites, structures, and behavior. Remarkably, the importance of experimental work, particularly the resolution of co-crystal structures for FDMs and their substrate complexes, cannot be understated. Such experimental data are crucial for validating computational predictions and gaining precise insights into how these enzymes function. Continuous research in this area will be vital for the development of improved FDMs mutants and expanding their utility in various biocatalytic applications. In addition, there is an urgent need for the development of more high-throughput screening approaches with broader applications for identifying effective FDM mutants.

Secondly, overexpression of essential genes in the endogenous cofactor recirculation pathways can enhance the efficiency of the catalytic pathway. However, it’s essential to consider that this method may negatively impact strain growth due to metabolic disturbances. Exploring alternative approaches, such as introducing new metabolic pathways, reconstructing and regulating original metabolic pathways to enhance cofactor regeneration, or utilizing biomimetic coenzymes, could provide solutions to mitigate these challenges [96]. Addressing these challenges will be crucial in advancing the field of FDM engineering and further improving the biosynthesis of hydroxylated aromatic compounds.

Lastly, protein scaffolding technology represents an innovative strategy in biosynthesis to boost the production of desired compounds [97, 98]. By immobilizing the monooxygenase and flavin reductase components of two-component FDMs onto a scaffold protein, it significantly reduces the spatial separation between these enzymes. This proximity may facilitate the efficient transfer of substrates and cofactors required for the hydroxylation reaction, resulting in a substantial enhancement of the catalytic efficiency of FDMs. With the ongoing advancements in computational and biological technologies, as well as a deeper understanding of the catalytic mechanisms of FDMs, we can anticipate the development of more efficient FDMs for the biosynthesis of valuable aromatic compounds.

## Data Availability

Not applicable.
